# Cucumber mosaic virus 2b proteins inhibit virus‐induced aphid resistance in tobacco

**DOI:** 10.1111/mpp.12892

**Published:** 2019-11-27

**Authors:** Trisna Tungadi, Ruairí Donnelly, Ling Qing, Javaid Iqbal, Alex M. Murphy, Adrienne E. Pate, Nik J. Cunniffe, John P. Carr

**Affiliations:** ^1^ Department of Plant Sciences University of Cambridge Downing Street Cambridge CB2 3EA UK; ^2^ College of Plant Protection Southwest University No. 2, Tiansheng Road Chongqing China

**Keywords:** host manipulation, jasmonate, non‐persistent, vector, viral suppressor of RNA silencing, virus transmission

## Abstract

Cucumber mosaic virus (CMV), which is vectored by aphids, has a tripartite RNA genome encoding five proteins. In tobacco (*Nicotiana tabacum*), a subgroup IA CMV strain, Fny‐CMV, increases plant susceptibility to aphid infestation but a viral mutant unable to express the 2b protein (Fny‐CMV∆2b) induces aphid resistance. We hypothesized that in tobacco, one or more of the four other Fny‐CMV gene products (the 1a or 2a replication proteins, the movement protein, or the coat protein) are potential aphid resistance elicitors, whilst the 2b protein counteracts induction of aphid resistance. Mutation of the Fny‐CMV 2b protein indicated that inhibition of virus‐induced resistance to aphids (*Myzus persicae*) depends on amino acid sequences known to control nucleus‐to‐cytoplasm shuttling. LS‐CMV (subgroup II) also increased susceptibility to aphid infestation but the LS‐CMV∆2b mutant did not induce aphid resistance. Using reassortant viruses comprising different combinations of LS and Fny genomic RNAs, we showed that Fny‐CMV RNA 1 but not LS‐CMV RNA 1 conditions aphid resistance in tobacco, suggesting that the Fny‐CMV 1a protein triggers resistance. However, the 2b proteins of both strains suppress aphid resistance, suggesting that the ability of 2b proteins to inhibit aphid resistance is conserved among divergent CMV strains.

## Graphical abstract

The cucumber mosaic virus 1a protein forms the replicase complex with protein 2a. In tobacco, 1a can elicit anti‐aphid resistance (antibiosis) but the viral 2b counterdefence protein prevents this.

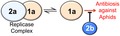

## Introduction

*Cucumber mosaic virus* (CMV) is the type species of the genus *Cucumovirus*. Strains of CMV can be classified into subgroups using RNA sequence data (Roossinck *et al.*, 1999). CMV has an extensive host range and is vectored by over 80 species of aphid (Jacquemond, 2012; Yoon *et al*., 2019). Aphids vector CMV in a nonpersistent manner, i.e. virus particles attach to receptors within the aphid stylet and are acquired and lost rapidly during short probes of the host epidermal cells (Hull, 2013; Krenz *et al.*, 2015). *Myzus persicae* (green peach or peach‐potato aphid) is a generalist herbivore that occurs worldwide, has a wide plant host range and is an efficient CMV vector (Devonshire *et al.*, 1998; Jacquemond, 2012; Louis and Shah, 2013; Nalam *et al.*, 2019; Pickett *et al.*, 1992; Yoon *et al*., 2019).

CMV has a tripartite, positive‐sense RNA genome that encodes five proteins (Jacquemond, 2012; Palukaitis and García‐Arenal, 2003; Yoon *et al*., 2019). RNA 1 is the translation template for the 1a protein, a replicase component with methyltransferase/helicase activity (Gal‐On *et al.*, 1994). RNA 2 is the translation template for the 2a protein, the viral RNA‐dependent RNA polymerase (Hayes and Buck, 1990). RNA 2 also encodes the 2b counterdefence protein, which is translated from a subgenomic viral mRNA (Ding *et al.*, 1994). RNA 3 encodes the cell‐to‐cell movement protein and the coat protein (CP) (Jacquemond, 2012; Palukaitis and García‐Arenal, 2003; Yoon *et al*., 2019). The CP controls acquisition of CMV particles by the aphid stylet and is therefore essential for vectored transmission (Gera *et al.*, 1979; Krenz *et al.*, 2015). The CMV 2b protein, in common with several other viral proteins that inhibit host RNA silencing, may influence CMV transmission indirectly by contributing to virus‐induced alterations in plant–aphid interactions (Westwood *et al.*, 2013, 2014; Wu *et al.*, 2017; Ziebell *et al.*, 2011).

Viruses alter interactions between plants and insect vectors in ways that may promote virus transmission, or which may foster persistence and spread of vectors in the environment (Casteel *et al*., 2014, 2015; Donnelly *et al.*, 2019; Groen *et al.*, 2017; Mauck, 2016). The effects of CMV on plant–aphid interactions are not the same for all plant hosts. For example, in cucurbits the subgroup IA CMV strain Fny (Fny‐CMV) induces changes in emission of plant volatile organic compounds that attract aphids to infected hosts (Carmo‐Souza *et al*., 2014; Mauck *et al.*, 2010). However, the quantitative and qualitative changes in volatile emission induced in tobacco (*Nicotiana tabacum*) plants by Fny‐CMV do not affect aphid behaviour (Tungadi *et al.*, 2017). Fny‐CMV induces production of distasteful substances in squash and *Arabidopsis thaliana* plants that inhibit prolonged feeding from the phloem (Mauck *et al.*, 2010; Westwood *et al.*, 2013). This effect is likely to enhance virus acquisition from the host’s epidermal cells and intensify transmission to nearby susceptible hosts (Donnelly *et al.*, 2019). In contrast, aphids placed on tobacco plants infected with Fny‐CMV exhibit increased feeding from the phloem, and aphids confined on these plants exhibit increased survival and reproduction (Ziebell *et al.*, 2011). Although virus‐induced susceptibility to aphid infestation will not promote rapid virus transmission to neighbouring hosts, it may increase aphid population density on infected plants and stimulate birth of winged aphids that could facilitate longer distance transmission and contribute to epidemic development at the landscape scale (Donnelly *et al.*, 2019).

Although *M. persicae* survival and reproduction is increased on tobacco plants infected with Fny‐CMV, aphids showed dramatic increases in mortality and decreased reproduction on plants infected with the mutant virus, Fny‐CMV∆2b (Ziebell *et al.*, 2011). Since Fny‐CMV∆2b cannot express the 2b protein we hypothesized that this factor inhibits induction of an antibiosis‐type resistance to aphids that is triggered by one or more of the other Fny‐CMV gene products, i.e. the CMV 1a or 2a replication proteins, the movement protein or the CP.

Tobacco (cv. Xanthi) plants were grown and inoculated with purified virions as described by Tungadi *et al. *(2017). Virions were propagated and purified (Lot *et al.*, 1972) from *N. benthamiana* plants previously inoculated with appropriate mixtures of *in vitro* synthesized synthetic viral RNAs for reconstitution of previously described wild‐type, mutant or reassortant viruses (Lewsey *et al.*, 2009; Owen and Palukaitis, 1988; Pita and Roossinck, 2014; Ryabov *et al.*, 2001; Soards *et al.*, 2002; Westwood *et al.*, 2013; Zhang *et al.*, 1994). Successful infection was confirmed using rapid immunodiagnostic kits (Agdia Inc., Elkhart, IN, USA) and quantified by double‐sandwich enzyme‐linked immunosorbent assay (DAS ELISA) (Bioreba AG, Reinach, Switzerland). Culturing conditions, clip caging on leaves of tobacco and monitoring of aphid reproduction for *M. persicae* (clone US1L: Devonshire and Sawicki, 1979) have been described by Ziebell *et al. *(2011). Statistical analyses were done using R software v. 3.5.0 and tables listing results for experiments are available in the Supporting Information (Tables [Link], [Link], [Link], [Link], [Link], [Link]) (Benjamini and Hochberg, 1995; Crawley, 2007; Hothorn *et al*., 2008).

**Figure 1:**
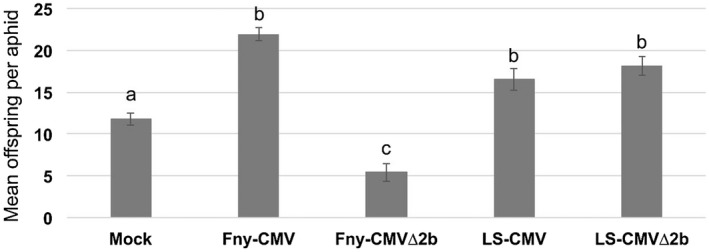
LS‐CMV∆2b infection does not induce aphid resistance in tobacco. Single 1‐day‐old aphids (*Myzus persicae*) were clip‐caged on each plant and 14 days later offspring numbers were recorded (*n* = 16 for each treatment group). Data marked with different letters (a, b, c and d) differ significantly (*P* < 0.05: FDR‐adjusted *P*‐value) (Table S2). Error bars indicate standard error around the mean. The experiment was performed five times and similar results were observed (Table S1). Data for offspring production and founder aphid survival for this experiment is available in Table S1 (labelled as ‘Experiment 5’). Statistical analysis is listed in Table S2 and described in the Supporting Information.

**Figure 2:**
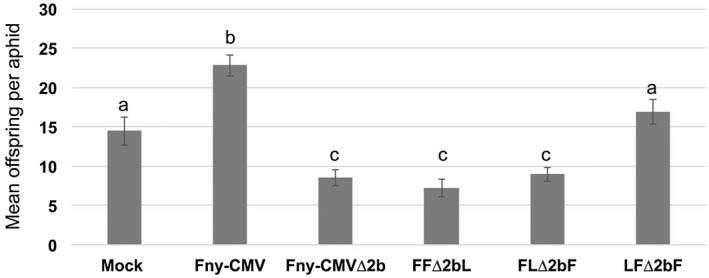
CMV RNA 1 or its gene product, the 1a protein, conditions resistance induction by Fny‐CMV∆2b against aphids. Reproduction of aphids (*Myzus persicae*) was decreased significantly on tobacco plants infected with the *2b* gene deletion mutant Fny‐CMV∆2b. This also occurred on plants infected with the reassortants FF∆2bL and FL∆2bF (which both have RNA 2 molecules that cannot express the 2b proteins encoded by Fny‐CMV or LS‐CMV, respectively, and which both possess Fny‐CMV RNA 1). Aphid performance was not diminished on plants infected with wild‐type Fny‐CMV or the reassortant LF∆2bF, which lacks the ability to express a 2b protein and has the RNA 1 of LS‐CMV. Single 1‐day‐old aphid nymphs were clip‐caged on each plant and their reproduction and survival were monitored for 14 days (*n* = 28 aphids per treatment group in this experiment). Data marked with different letters (a, b and c) differ significantly (*P* < 0.05, FDR‐adjusted *P*‐value). Error bars indicate standard error around the mean. The experiment was done four times independently with similar results seen each time (Table S3). Data for aphid survival and total number of offspring in this experiment can be found in Table S3 (labelled as ‘Experiment 2’). Statistical analysis is listed in Table S4 and described in the Supporting Information.

**Figure 3:**
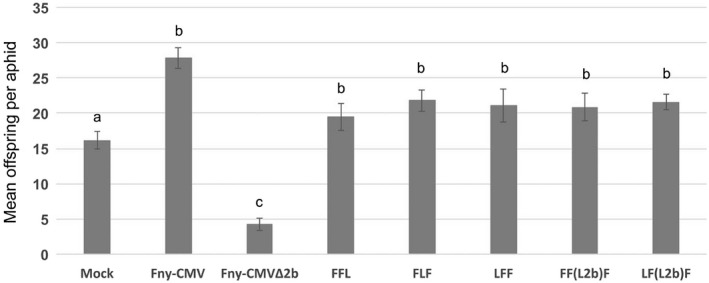
The ability of 2b proteins to inhibit induction of aphid resistance is conserved between the two CMV strains, Fny‐CMV (subgroup IA) and LS‐CMV (subgroup II). Substituting Fny 2b protein with LS 2b protein and vice versa did not lead to a decrease in aphid reproduction. Aphid (*Myzus persicae*) reproduction was not significantly depressed on tobacco plants infected with reassortants FF(L2b)F, LF(L2b)F, FFL, FLF and LFF when compared to aphids placed on wild‐type Fny‐CMV‐infected plants. As expected, aphid reproduction was significantly suppressed on plants infected with Fny‐CMV∆2b compared to aphids on mock‐inoculated control plants, Fny‐CMV‐infected plants and on plants infected with reassortants with intact RNA 2 molecules from either the Fny‐ or LS‐strain of CMV. Single 1‐day‐old aphid nymphs (founder aphids) were clip‐caged on each plant and their reproduction and survival monitored for 14 days. Data for mean offspring per founder aphid is presented here (*n* = 12 aphids per treatment in this experiment). Data marked with different letters (a, b and c) differ significantly (*P < *0.05, FDR‐adjusted *P*‐value). Error bars indicate standard error around the mean. The experiment has been done three times independently with similar results (Table S5). Data for founder aphid survival and total offspring in this experiment can be found in Table S5 (labelled as ‘Experiment 3’). Statistical significance is listed in Table S6 and described in the Supporting Information.

We examined the effects that Fny‐CMV and LS‐CMV have on aphid–host interactions in tobacco using aphid reproduction and survival as measures of virus‐induced changes in aphid performance (Fig. 1, Tables S1 and S2). Single 1‐day‐old aphid nymphs (colony ‘founders’; Table S1) were clip‐caged individually on leaves of mock‐inoculated or virus‐infected plants and their offspring counted 14 days later. Reproduction of *M. persicae* was enhanced on plants infected with wild‐type Fny‐CMV and LS‐CMV compared to aphids on mock‐inoculated control plants (Fig. 1). On tobacco plants infected with Fny‐CMVΔ2b aphid reproduction was significantly decreased compared to aphids on Fny‐CMV‐infected plants or on mock‐inoculated control plants (Tables S1 and S2). In four out of five experiments (Table S1), the survival of founder aphids on Fny‐CMVΔ2b‐infected plants was decreased compared to founder aphids placed on Fny‐CMV‐infected plants. This is consistent with previous findings (Ziebell *et al.*, 2011). In contrast, LS‐CMVΔ2b did not induce antibiosis (reduction in aphid survival and reproduction) against aphids on tobacco when compared to aphids on Fny‐CMV and LS‐CMV‐infected tobacco plants (Fig. 1, Tables S1 and S2). Here, and in subsequent experiments, it was noted that aphid reproduction and survival were in some cases improved on plants infected with wild‐type Fny‐CMV, LS‐CMV, or with LS‐CMVΔ2b when compared to aphids on mock‐inoculated control plants, although this trend was not statistically significant in all cases (Figs 1, 2, 3, Tables [Link], [Link], [Link], [Link], [Link], [Link]).

These strain‐specific differences between the effects of Fny‐CMV and LS‐CMV on host–aphid interactions (Fig. 1, Tables S1 and S2) enabled us to identify CMV genomic RNAs and corresponding gene products using reassortant (pseudorecombinant) viruses (Rao and Francki, 1982; Westwood *et al.*, 2013; Zhang *et al.*, 1994). These comprised mixtures of *in vitro* synthesized genomic RNAs 1, 2 and 3 derived from wild‐type LS‐CMV or Fny‐CMV, or mixtures including mutant Fny‐CMV or LS‐CMV RNA 2 molecules unable to express the 2b protein (Fig. 2, Tables S3 and S4). There were marked decreases in founder aphid survival and reproduction on plants infected with Fny‐CMVΔ2b compared to aphids on mock‐inoculated and Fny‐CMV‐infected plants (consistent with previous results; Ziebell *et al.*, 2011). Similar decreases in aphid reproduction were seen on tobacco plants infected with the reassortant FF∆2bL (which was reconstituted from Fny‐CMV RNA 1, LS‐CMV RNA 3 and the Δ2b mutant of Fny‐CMV RNA 2) and with the reassortant FLΔ2bF (which was reconstituted from wild‐type Fny‐CMV RNAs 1 and 3, and the Δ2b mutant of LS‐CMV RNA 2) (Fig. 2, Tables S3 and S4). However, aphid reproduction was not significantly impaired on tobacco plants infected with the reassortant LFΔ2bF, which was reconstituted from the Δ2b mutant of Fny‐CMV RNA 2 with wild‐type RNAs 1 and 3 from LS‐CMV and Fny‐CMV, respectively (Fig. 2, Tables S3 and S4). The results indicate that neither Fny‐CMV RNA 3, nor the two proteins it encodes (movement protein and CP), play significant roles in aphid resistance induction during infection in tobacco. The results indicate that the RNA 1 of Fny‐CMV conditions aphid resistance induction in plants infected with Fny‐CMVΔ2b.

CMV RNA 1 is monocistronic and is the translation template for only one viral gene product, the 1a protein (Jacquemond, 2012; Palukaitis and García‐Arenal, 2003; Yoon *et al*., 2019). Thus, we think it most probable that the Fny‐CMV 1a protein can trigger antibiosis against aphids in tobacco, while the LS‐CMV 1a protein cannot do this. Although this is the most likely and straightforward explanation, we cannot rule out entirely the possibility that differences in RNA sequence or secondary structure between the RNAs 1 of LS‐CMV and Fny‐CMV may be responsible. The CMV 1a protein is already known to have multiple biological functions. The 1a protein assembles with the 2a protein and host factors into the viral replicase complex (Cillo *et al.*, 2002; Hayes and Buck, 1990; Nitta *et al.*, 1988), and it also influences viral systemic movement in the plant and symptom severity (Gal‐On *et al.*, 1994; Mochizuki and Ohki, 2012). The 1a protein also affects resistance and host range. For example, it triggers a hypersensitive response against Ns‐CMV in *N. glutinosa* (Deveki *et al*., 2004), the 5′ nontranslated sequence of RNA 1 plus amino acids 485–826 of the 1a protein condition infection of lily by HL‐CMV (Yamaguchi *et al.*, 2005), and breakage of resistance due to the *Capsicum Cmr1* gene by P1‐CMV involves changes at amino acids 865, 896, 957 and 980 of the 1a protein (Kang *et al.*, 2012). We suggest that the ability to induce resistance to aphids in tobacco is an additional RNA 1/1a protein activity, albeit one that is deleterious to transmission by these insect vectors. It is plausible, therefore, that the 2b protein does somehow counteract this activity during infection of tobacco.

To further investigate the strain‐specific differences between Fny‐CMV and LS‐CMV in their ability to affect host–aphid interactions in tobacco, we made use of a recombinant CMV RNA 2 molecule that encodes the Fny‐CMV 2a protein and the LS‐CMV 2b protein developed by Pita and Roossinck (2014) and referred to here as RNA2 F(L2b). Assessment of the effects of reassortant viruses on aphid performance on tobacco indicated that the presence of RNA 2 from Fny‐CMV or LS‐CMV was sufficient to prevent induction of aphid resistance by the Fny‐CMV 1a protein or the Fny‐CMV RNA 1 molecule (Fig. 3, Tables S5 and S6). The work of Ziebell *et al. *(2011) and data presented here (Figs 1, 2, 3) showed that the Fny‐CMV 2b protein can inhibit the induction of aphid resistance. The amino acid sequences of the Fny‐CMV and LS‐CMV 2b proteins display differences that are characteristic of the CMV subgroups to which these strains belong (IA and II, respectively) (Mayers *et al.*, 2000). However, the results obtained here with reassortant viruses that possess the recombinant RNA2 F(L2b) show that the 2b protein of LS‐CMV is capable of inhibiting aphid resistance induction by the Fny‐CMV 1a protein (Fig. 3, Tables S5 and S6). This indicates that this property is a conserved function of all 2b proteins. Although the LS‐CMV 1a protein does not induce resistance to aphids in tobacco (Figs 1 and 2), it is possible that it might do so in other hosts and this may explain why the LS‐CMV 2b protein can inhibit aphid resistance induced by the 1a protein of Fny‐CMV. The virus accumulation levels varied for the various strains and reassortants (Fig. S1), as did disease symptom severity (Fig. S2). However, there appeared to be no obvious relationship between either virus titre or symptom severity and aphid resistance induced by RNA 1 or the 1a protein.

The CMV 2b protein can be found both in the nucleus and the cytoplasm of the host plant. It has been shown that the proportion of 2b protein that accumulates in the nucleus compared to the cytoplasm profoundly affects symptom expression during viral infection. In addition, the level of 2b protein accumulation in these compartments modulates the effects of the 2b protein on priming of salicylic acid biosynthesis, antiviral RNA silencing and microRNA‐regulated gene expression (Cillo *et al.*, 2009; Du *et al.*, 2014; González *et al.*, 2010, 2012; Lewsey *et al*., 2007; Zhou *et al.*, 2014). We regenerated Fny‐CMV variants carrying mutations in the *2b* gene sequence that affect protein domains controlling nuclear/cytoplasmic partitioning (Lewsey *et al.*, 2009), and used them to investigate if inhibiting nucleus‐to‐cytoplasm shuttling of the 2b protein affects the ability of the 2b protein to counteract RNA 1/1a protein‐induced aphid resistance in tobacco.

The 2b domain‐specific mutants, which carried 2b protein‐coding sequences with deletions corresponding to either one or both of the nuclear localization sequences (mutants Fny‐CMV∆NLS1, Fny‐CMV∆NLS2 or Fny‐CMV∆NLS1 + 2) or to deletion of the KSPSE domain (Fny‐CMV∆KSPSE), have been described previously (Lewsey *et al.*, 2009). The two nuclear localization sequence domains of the 2b protein sequence are conserved among CMV subgroup IA strains and are required for entry of the protein into the nucleus and for RNA silencing suppressor activity (Du *et al.*, 2014; González *et al.*, 2010, 2012; Goto *et al*., 2007; Mayers *et al.*, 2000; Wang *et al.*, 2004). The KSPSE protein domain (comprising the amino acid residues lysine‐serine‐proline‐serine‐glutamic acid) can be reversibly phosphorylated and is required for shuttling of the 2b protein between the nuclear and cytoplasmic compartments of the cell (Lewsey *et al.*, 2009; Nemes *et al.*, 2017). Deletion of this sequence results in a 2b protein variant that appears to be trapped in the nucleolus (González *et al.*, 2010).

**Figure 4:**
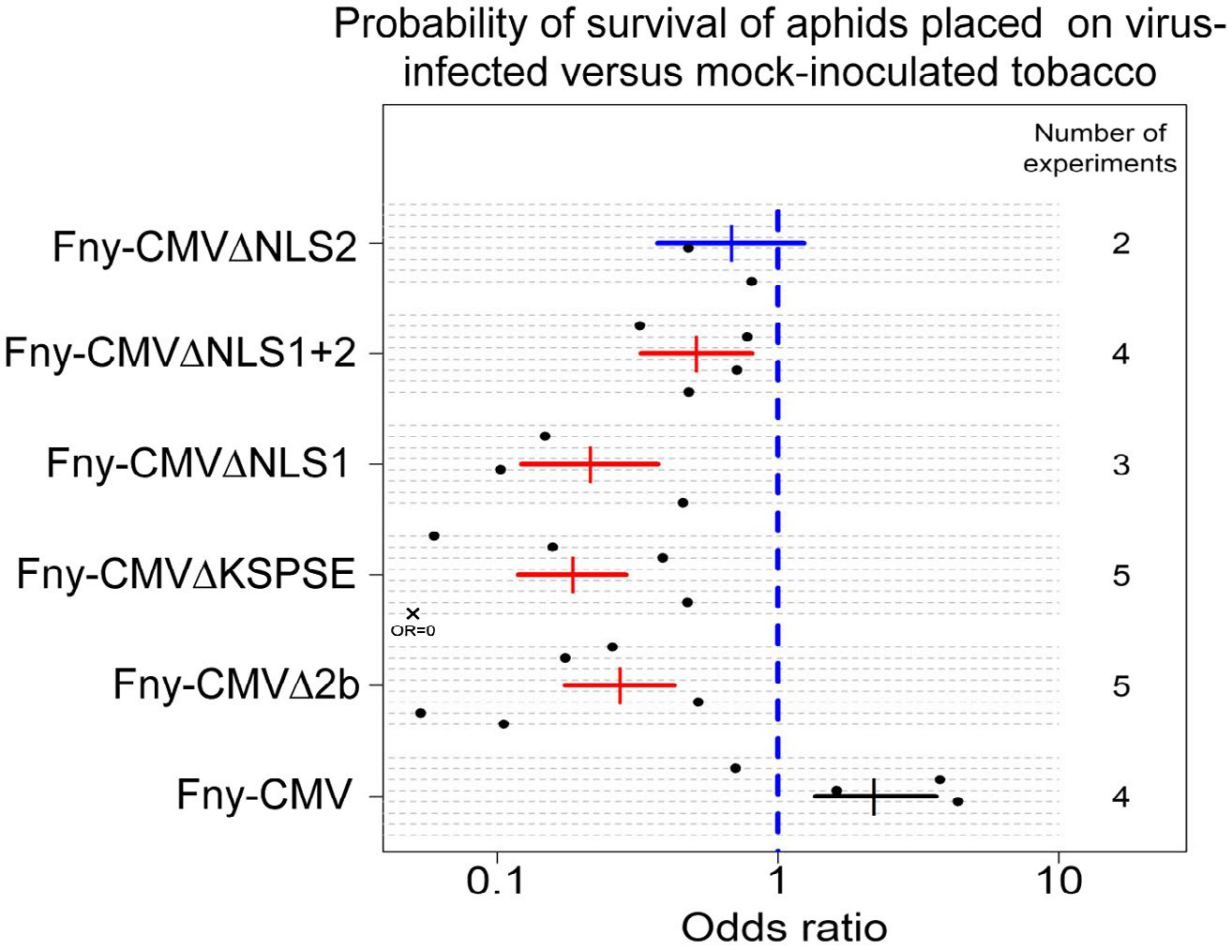
Decreased survival of aphids on tobacco plants infected with the mutant viruses Fny‐CMV∆NLS1 + 2, Fny‐CMV∆NLS1, Fny‐CMV∆NLS2 and Fny‐CMV∆KSPSE. Five 1‐day‐old *Myzus persicae* nymphs were confined individually in clip cages attached to the abaxial surface of the leaves of each tobacco plant for 11 days. Ten plants were used per treatment, i.e. *n* = 50 aphids per treatment. All plants were inoculated with wild‐type Fny‐CMV, with the viral mutants indicated, or were mock‐inoculated with water 14 days prior to placement of aphids. The solid lines for each treatment show the 95% confidence interval on the estimated odds ratio of aphid survival relative to mock‐inoculated plants. The solid black points show the odds ratio relative to mock‐inoculated as measured in individual experiments (a total of eight experiments were performed involving different combinations of treatments). To interpret this presentation of the data, note that the ‘odds’ of an event that occurs with probability *p* is simply *p*⁄(1 − *p*), therefore if the probability of survival on mock‐inoculated plants in a particular experiment is *p*_m_, and the probability of survival under a particular treatment in the same experiment is *p*_t_, then the odds ratio for that treatment in that experiment is [*p*_t_⁄(1 − *p*_t_)]⁄[ *p*_m_⁄(1 − *p*_m_)]. The 95% confidence interval on the estimated odds ratio did not overlap 1.0 for all treatments except Fny‐CMV∆NLS2. Thus, all treatments apart from Fny‐CMV∆NLS2 had a statistically significant effect on aphid survival relative to mock‐inoculated. No aphid survived after 11 days on the Fny‐CMV∆KSPSE inoculated plants in Experiment 1. This value falls outside the range of values of the (logarithmic) odds ratio and is marked with a cross. A detailed technical explanation of the statistical analysis can be found in the Supplementary Information.

Figure 4 shows data combined from multiple experiments monitoring aphid survival. The aphids were caged on tobacco plants that, 14 days earlier, had been infected with wild‐type Fny‐CMV or with the indicated viral mutants, or that had been mock‐inoculated with water. Five 1‐day‐old *M. persicae* nymphs were confined individually in clip cages on the abaxial surface of the leaves of each plant for 11 days. Ten plants were used per treatment, i.e. *n* = 50 aphids per treatment. To interpret Fig. 4 note that lower proportions of aphids surviving after 11 days under a particular treatment than on the mock‐inoculated plants corresponds to a relative odds ratio (*x*‐axis) that is lower than 1, and higher survival corresponds to a relative odds ratio larger than 1 (see Supporting Information). The 95% confidence interval on the estimated odds ratio did not overlap 1.0 for all treatments except for aphids placed on plants infected with the mutant Fny‐CMV∆NLS2. Thus, all virus treatments apart from Fny‐CMV∆NLS2 and wild‐type Fny‐CMV caused statistically significant decreases in the odds of aphid survival relative to survival of aphids on mock‐inoculated plants. It appears that mutations that inhibit either entry of the 2b protein into the host cell nucleus (mutants Fny‐CMV∆NLS1, Fny‐CMV∆NLS2 or Fny‐CMV∆NLS1 + 2), or its exit from the nuclear compartment (Fny‐CMV∆KSPSE), compromise the ability of the 2b protein to prevent 1a‐induced aphid resistance in tobacco (Fig. 4). This indicates that the ability of the 2b protein to move freely between these cellular compartments must be necessary in some way for it to be able to counter the aphid resistance‐inducing activity of Fny‐CMV RNA 1/the 1a protein.

In conclusion, CMV 2b proteins possess the ability to inhibit the induction of antibiosis by the Fny‐CMV RNA 1 and/or the 1a protein and this is conserved between 2b proteins of CMV strain Fny from subgroup IA and CMV strain LS from subgroup II. It remains to be seen whether this ability to prevent the induction of resistance to aphids is solely due to the ability of CMV 2b proteins to inhibit jasmonate‐dependent defensive signalling, which is an important element of plant anti‐insect defence (Lewsey *et al.*, 2010; Westwood *et al.*, 2014). Interestingly, aphids survived and reproduced well on tobacco plants infected with either wild‐type LS‐CMV or with the *2b* gene deletion mutant, LS‐CMVΔ2b. These data suggest that the LS‐CMV 2b protein is not the only gene product encoded by this CMV strain that induces susceptibility of tobacco to aphid infestation. Experiments with reassortant viruses made up of genomic RNAs from Fny‐CMV and LS‐CMV showed that Fny‐CMV RNA 1 encodes the elicitor of the strong resistance to aphids hypothesized by Ziebell *et al. *(2011). However, the Fny‐CMV 1a protein’s previously documented effect on host–aphid interactions, in *A. thaliana*, contrasts markedly with its effect in tobacco, as does the effect of the 2b protein. In *A. thaliana*, it is the Fny‐CMV 2b protein that induces antibiosis against aphids while the 1a protein is the factor that prevents 2b‐induced antibiosis induction (Westwood *et al.*, 2013). In both plant hosts, the 1a and 2b proteins appear to have antagonistic roles in conditioning CMV‐induced effects on aphid–plant interactions suggesting the interplay of the 1a and 2b proteins in some way determines the outcome (induction of aphid resistance or aphid susceptibility) of CMV infection on plant–aphid interactions in different hosts. This reinforces previous work showing that the effects of viral proteins on plant–aphid interactions are complex and combinatorial (Westwood *et al.*, 2013, 2014).

## Supporting information


**Fig. S1** Accumulation of wild‐type Fny‐CMV and LS‐CMV and mutant or reassortant viruses in tobacco. At 14 days post‐inoculation the accumulation of coat protein was measured in systemicallyinfected leaves using DAS ELISA for plants that had been mock‐inoculated or infected with the wildtype, reassortant or mutant viruses indicated. A405 values were corrected by subtraction of the value for mock‐inoculated plants in each experiment. (A) For each treatment group: *n* = 3 for mockinoculated plants, and plants infected with Fny‐CMV or LS‐CMV, *n* = 6 for plants infected with Fny‐CMVΔ2b and LS‐CMVΔ2b, respectively. (B) For each treatment group: *n* = 3 for mock‐inoculated plants, *n* = 10 for Fny‐CMV, Fny‐CMVΔ2b, FF(L2b)F, FLF, and FFL‐infected plants respectively, *n* = 9 for LF(L2b)F and LFF‐infected plants, respectively. (C) For each treatment group: *n* = 3 for mockinoculated plants, *n* = 3 for plants infected with Fny‐CMV, and *n* = 6 for Fny‐CMVΔ2b, FFΔ2bL, LFΔ2bF and FLΔ2bF‐infected plants respectively.

**Fig. S2** Typical symptoms induced in tobacco by wild‐type Fny‐CMV and LS‐CMV and indicated mutant and reassortant viruses. Plants were inoculated or mock‐inoculated 14–21 days prior to photography. Scale bars indicate 3 cm.

**Table S1** Aphid performance on tobacco plants infected with wild‐type Fny‐CMV, LS‐CMV or their corresponding *2b* gene deletion mutants.

**Table S2** Statistical analysis on aphid reproduction on tobacco plants infected with wild‐type Fny‐CMV, LS‐CMV or their corresponding *2b* gene deletion mutants.

**Table S3** Aphid performance on tobacco plants infected with wild‐type Fny‐CMV, Fny‐CMVΔ2b LS‐CMV and reassortant viruses lacking the ability to express the *2b* gene.

**Table S4** Statistical analysis on aphid reproduction on tobacco plants infected with wild‐type Fny‐CMV, Fny‐CMVΔ2b LS‐CMV and reassortant viruses lacking the ability to express the *2b* gene.

**Table S5** Aphid performance on tobacco plants infected with Fny‐CMV, Fny‐CMVΔ2b LS‐CMV, selected reassortant viruses including those constituted using a recombinant Fny‐CMV RNA 2 expressing the LS‐CMV *2b* gene sequence.

**Table S6** Statistical analysis for aphid reproduction on tobacco plants infected with Fny‐CMV, Fny‐CMVΔ2b LS‐CMV, selected reassortant viruses including those constituted using a recombinant Fny‐CMV RNA 2 expressing the LS‐CMV *2b* gene sequence.

**Methods S1** Supplementary Methods.

## Data Availability

The data that support the findings of this study are available from the corresponding author on reasonable request.
