# Supplementary material for: Cucumber mosaic virus 2b proteins inhibit virus‐induced aphid resistance in tobacco
Source: Mol Plant Pathol. 2019 Nov 27;21(2):250–7. doi: 10.1111/mpp.12892 (PMC6988427; doi:10.1111/mpp.12892)
Supplement: Supplementary file 1 — Fig. S1 Accumulation of wild‐type Fny‐CMV and LS‐CMV and mutant or reassortant viruses in tobacco. At 14 days post‐inoculation the accumulation of coat protein was measured in systemicallyinfected leaves using DAS ELISA for plants that had been mock‐inoculated or infected with the wildtype, reassortant or mutant viruses indicated. A405 values were corrected by subtraction of the value for mock‐inoculated plants in each experiment. (A) For each treatment group: n = 3 for mockinoculated plants, and plants infected with Fny‐CMV or LS‐CMV, n = 6 for plants infected with Fny‐CMVΔ2b and LS‐CMVΔ2b, respectively. (B) For each treatment group: n = 3 for mock‐inoculated plants, n = 10 for Fny‐CMV, Fny‐CMVΔ2b, FF(L2b)F, FLF, and FFL‐infected plants respectively, n = 9 for LF(L2b)F and LFF‐infected plants, respectively. (C) For each treatment group: n = 3 for mockinoculated plants, n = 3 for plants infected with Fny‐CMV, and n = 6 for Fny‐CMVΔ2b, FFΔ2bL, LFΔ2bF and FLΔ2bF‐infected plants respectively. [file MPP-21-250-s001.pdf]

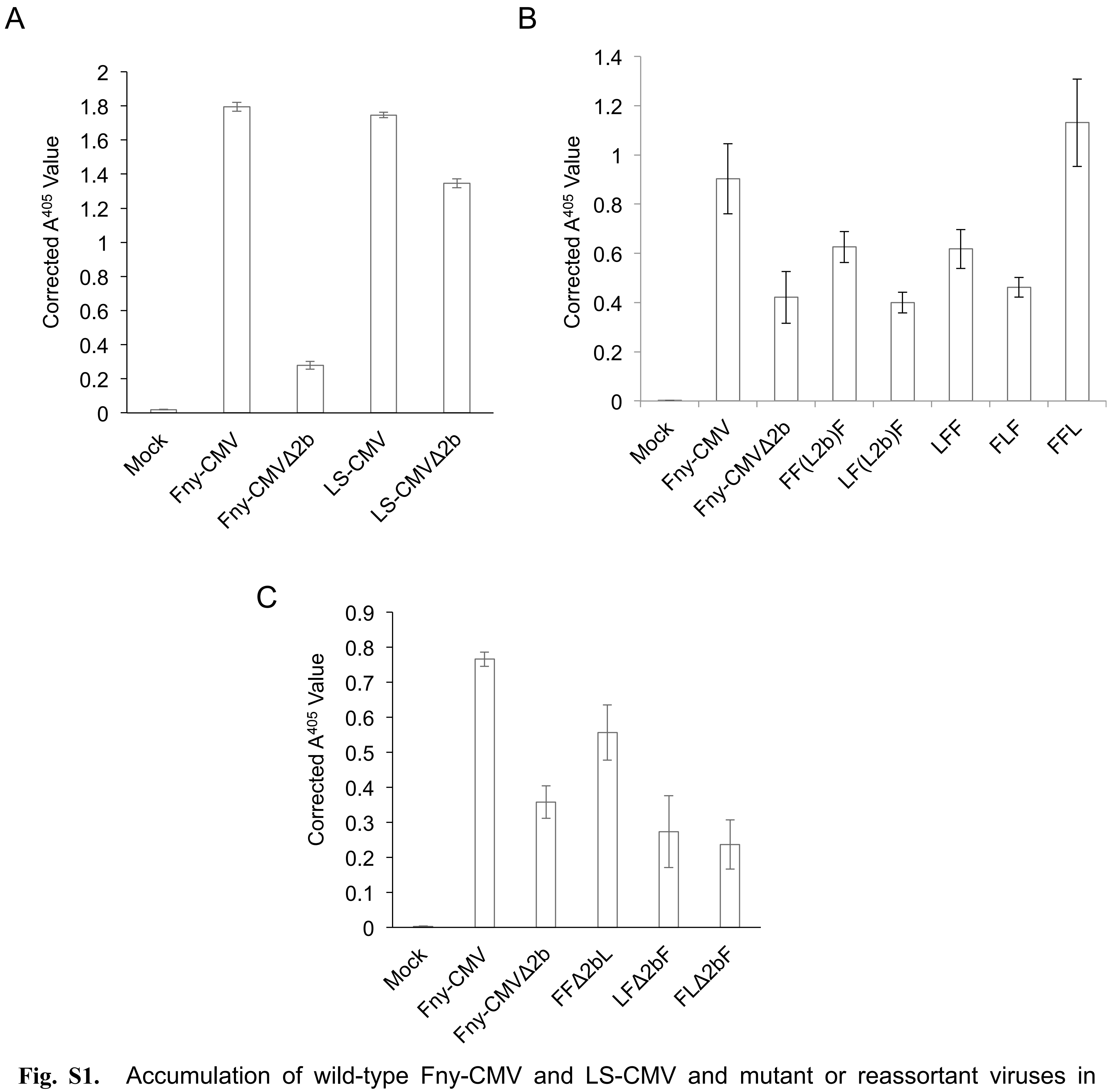

Fig. S1. Accumulation of wild-type Fny-CMV and LS-CMV and mutant or reassortant viruses in tobacco. At 14 days post-inoculation the accumulation of coat protein was measured in systemically-infected leaves using DAS ELISA for plants that had been mock-inoculated or infected with the wild-type, reassortant or mutant viruses indicated. A405 values were corrected by subtraction of the value for mock-inoculated plants in each experiment. (A) For each treatment group: n = 3 for mock-inoculated plants, and plants infected with Fny-CMV or LS-CMV, n = 6 for plants infected with Fny-CMVΔ2b and LS-CMVΔ2b, respectively. (B) For each treatment group: n = 3 for mock-inoculated plants, n = 10 for Fny-CMV, Fny-CMVΔ2b, FF(L2b)F, FLF, and FFL-infected plants respectively, n = 9 for LF(L2b)F and LFF-infected plants, respectively. (C) For each treatment group: n = 3 for mock-inoculated plants, n = 3 for plants infected with Fny-CMV, and n = 6 for Fny-CMVΔ2b, FFΔ2bL, LFΔ2bF and FLΔ2bF-infected plants respectively.
