# Supplementary material for: Cucumber mosaic virus 2b proteins inhibit virus‐induced aphid resistance in tobacco
Source: Mol Plant Pathol. 2019 Nov 27;21(2):250–7. doi: 10.1111/mpp.12892 (PMC6988427; doi:10.1111/mpp.12892)
Supplement: Supplementary file 2 — Fig. S2 Typical symptoms induced in tobacco by wild‐type Fny‐CMV and LS‐CMV and indicated mutant and reassortant viruses. Plants were inoculated or mock‐inoculated 14–21 days prior to photography. Scale bars indicate 3 cm. [file MPP-21-250-s002.pdf]

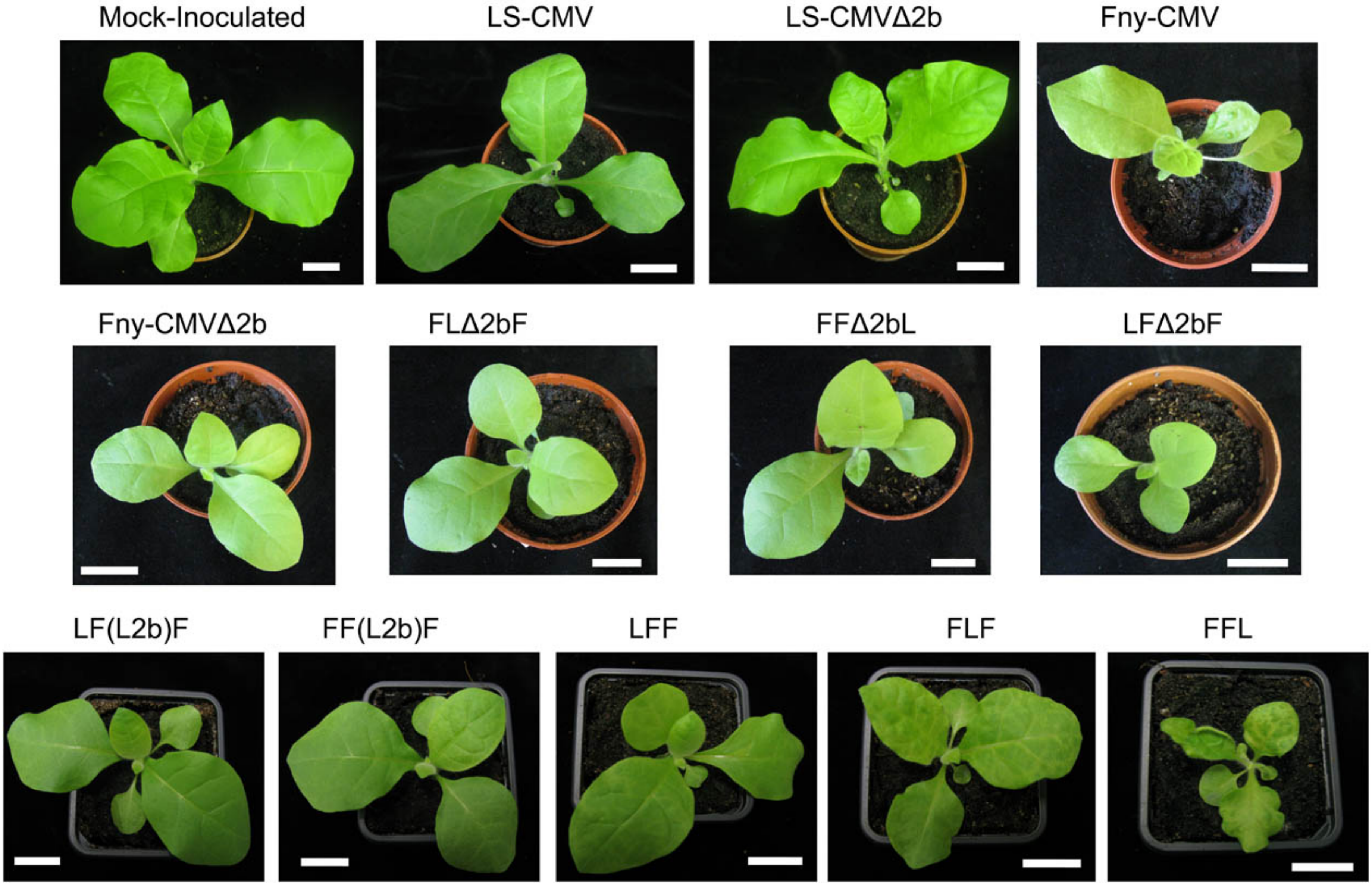

Fig. S2. Typical symptoms induced in tobacco by wild-type Fny-CMV and LS-CMV and indicated mutant and reassortant viruses. Plants were inoculated or mock-inoculated 14-21 days prior to photography. Scale bars indicate 3 cm.
