# Supplementary material for: Cucumber mosaic virus 2b proteins inhibit virus‐induced aphid resistance in tobacco
Source: Mol Plant Pathol. 2019 Nov 27;21(2):250–7. doi: 10.1111/mpp.12892 (PMC6988427; doi:10.1111/mpp.12892)
Supplement: Supplementary file 3 — Table S1 Aphid performance on tobacco plants infected with wild‐type Fny‐CMV, LS‐CMV or their corresponding 2b gene deletion mutants. [file MPP-21-250-s003.docx]

**Table S1.** Aphid performance on tobacco plants infected with wild-type Fny-CMV, LS-CMV or their corresponding *2b* gene deletion mutants

| **A. Number of surviving founder aphids at 14 dpi** | | | | | |
| --- | --- | --- | --- | --- | --- |
| **Treatment** | **Exp 1 (n=12)** | **Exp 2 (n=12)** | **Exp 3 (n=12)** | **Exp 4 (n=16)** | **Exp 5 (n=16)** |
| Mock-Inoculated | 8/12 | 7/12 | 7/12 | 16/16 | 16/16 |
| Fny-CMV | 9/12 | 10/12 | 9/12 | 16/16 | 16/16 |
| Fny-CMV∆2b | 5/12 | 4/12 | 9/12 | 13/16 | 14/16 |
| LS-CMV | 8/12 | 12/12 | 9/12 | 16/16 | 16/16 |
| LS-CMV∆2b | 11/12 | 10/12 | 9/12 | 16/16 | 16/16 |
| **B. Total aphid offspring at 14 dpi** | | | | | |
| Mock-Inoculated | 110 | 39 | 90 | 189 | 188 |
| Fny-CMV | 138 | 126 | 113 | 297 | 351 |
| Fny-CMV∆2b | 37 | 85 | 56 | 58 | 87 |
| LS-CMV | 159 | 153 | 119 | 323 | 264 |
| LS-CMV∆2b | 179 | 183 | 117 | 344 | 290 |
| **C. Mean offspring per founder aphid at 14 dpi** | | | | | |
| Mock-Inoculated | 9.2 | 3.3 | 7.5 | 11.8 | 11.8 |
| Fny-CMV | 11.5 | 10.5 | 9.4 | 18.6 | 21.9 |
| Fny-CMV∆2b | 3.1 | 7.1 | 4.7 | 3.6 | 5.4 |
| LS-CMV | 13.3 | 12.8 | 9.9 | 20.2 | 16.5 |
| LS-CMV∆2b | 14.9 | 15.3 | 9.8 | 21.5 | 18.1 |

**Notes.** Aphid reproduction and survival data from five independent experiments. Statistical analysis for aphid reproduction is described in Table S2 below. Data from Experiment 5 are displayed as a bar chart in Fig. 1
