# Supplementary material for: Cucumber mosaic virus 2b proteins inhibit virus‐induced aphid resistance in tobacco
Source: Mol Plant Pathol. 2019 Nov 27;21(2):250–7. doi: 10.1111/mpp.12892 (PMC6988427; doi:10.1111/mpp.12892)
Supplement: Supplementary file 4 — Table S2 Statistical analysis on aphid reproduction on tobacco plants infected with wild‐type Fny‐CMV, LS‐CMV or their corresponding 2b gene deletion mutants. [file MPP-21-250-s004.docx]

**Table S2.** Statistical analysis on aphid reproduction on tobacco plants infected with wild-type Fny-CMV, LS-CMV or their corresponding *2b* gene deletion mutants

| **Experiment 1** | Total aphid offspring | Mock | Fny-CMV | Fny-CMV∆2b | LS-CMV | LS-CMV∆2b |
| --- | --- | --- | --- | --- | --- | --- |
|  |  | 110 | 138 | 37 | 159 | 179 |
| Mock | 110 |  |  |  |  |  |
| Fny-CMV | 138 |  |  |  |  |  |
| Fny-CMV∆2b | 37 |  | * |  |  |  |
| LS-CMV | 159 |  |  | * |  |  |
| LS-CMV∆2b | 179 |  |  | * |  |  |
|  | | | | | | |
| **Experiment 2** | Total aphid offspring | Mock | Fny-CMV | Fny-CMV∆2b | LS-CMV | LS-CMV∆2b |
|  |  | 39 | 126 | 85 | 153 | 183 |
| Mock | 39 |  |  |  |  |  |
| Fny-CMV | 126 |  |  |  |  |  |
| Fny-CMV∆2b | 85 |  |  |  |  |  |
| LS-CMV | 153 |  |  |  |  |  |
| LS-CMV∆2b | 183 | * |  |  |  |  |
|  | | | | | | |
| **Experiment 3** | Total aphid offspring | Mock | Fny-CMV | Fny-CMV∆2b | LS-CMV | LS-CMV∆2b |
|  |  | 90 | 113 | 56 | 119 | 117 |
| Mock | 90 |  |  |  |  |  |
| Fny-CMV | 113 |  |  |  |  |  |
| Fny-CMV∆2b | 56 | * | * |  |  |  |
| LS-CMV | 119 |  |  | * |  |  |
| LS-CMV∆2b | 117 |  |  | * |  |  |
|  | | | | | | |
| **Experiment 4** | Total aphid offpsring | Mock | Fny-CMV | Fny-CMV∆2b | LS-CMV | LS-CMV∆2b |
|  |  | 189 | 297 | 58 | 323 | 344 |
| Mock | 189 |  |  |  |  |  |
| Fny-CMV | 297 | * |  |  |  |  |
| Fny-CMV∆2b | 58 | * | * |  |  |  |
| LS-CMV | 323 | * |  | * |  |  |
| LS-CMV∆2b | 344 | * |  | * |  |  |
|  | | | | | | |
| **Experiment 5** | Total aphid offspring | Mock | Fny-CMV | Fny- CMV∆2b | LS-CMV | LS-CMV∆2b |
|  |  | 188 | 351 | 87 | 264 | 290 |
| Mock | 188 |  |  |  |  |  |
| Fny-CMV | 351 | * |  |  |  |  |
| Fny-CMV∆2b | 87 | * | * |  |  |  |
| LS-CMV | 264 | * | * | * |  |  |
| LS-CMV∆2b | 290 | * | * | * |  |  |
|  | | | | | | |
| Across all experiments | Total aphid offspring | Mock | Fny-CMV | Fny- CMV∆2b | LS-CMV | LS-CMV∆2b |
|  |  |  |  |  |  |  |
| Mock |  |  |  |  |  |  |
| Fny-CMV |  | * |  |  |  |  |
| Fny-CMV∆2b |  | * | * |  |  |  |
| LS-CMV |  | * |  | * |  |  |
| LS-CMV∆2b |  | * |  | * |  |  |

**Notes.** Negative binomial regression (as explained above) were used to analyse statistical significance for pairwise comparisons made between treatments on aphid reproduction. Pairwise comparison between treatments marked with * denotes significance at *p* < 0.05 with FDR-adjusted *p-*value.

Aphids (*Myzus persicae*) confined on tobacco plants infected with Fny-CMV∆2b gave rise to significantly fewer offspring compared to aphids placed on plants under other treatment conditions (mock-inoculated, infected with Fny-CMV, LS-CMV or LS-CMV∆2b) in four out of five experiments. Note that reproduction of aphids on mock-inoculated plants was atypically low in Experiment 2 (compare results in other experiments here and with those in Tables S2 and S3).

Aphids confined on LS-CMV and LS-CMV∆2b infected plants gave rise to significantly more offspring compared to aphids placed on mock infected plants in three out of five experiments (Experiment 2, 4 and 5).

Lastly, aphids confined on Fny-CMV infected plants gave rise to significantly more offspring compared to aphids placed on mock-infected plants in two out of five experiments (Experiment 4 and 5). Data from Experiment 5 are displayed as a bar chart in Fig. 1.
