# Supplementary material for: Cucumber mosaic virus 2b proteins inhibit virus‐induced aphid resistance in tobacco
Source: Mol Plant Pathol. 2019 Nov 27;21(2):250–7. doi: 10.1111/mpp.12892 (PMC6988427; doi:10.1111/mpp.12892)
Supplement: Supplementary file 5 — Table S3 Aphid performance on tobacco plants infected with wild‐type Fny‐CMV, Fny‐CMVΔ2b LS‐CMV and reassortant viruses lacking the ability to express the 2b gene. [file MPP-21-250-s005.docx]

**Table S3.** Aphid performance on tobacco plants infected with wild-type Fny-CMV, Fny-CMV∆2b LS-CMV and reassortant viruses lacking the ability to express the *2b* gene.

| **A. Number of surviving founder aphids at 14 dpi** | | | | |
| --- | --- | --- | --- | --- |
| **Treatment** | **Exp 1 (n=22)** | **Exp 2 (n=28)** | **Exp 3 (n=16)** | **Exp 4 (n=14)** |
| Mock-Inoculated | 19/22 | 25/28 | 16/16 | 14/14 |
| Fny-CMV | 22/22 | 28/28 | 16/16 | 14/14 |
| Fny-CMV∆2b | 10/22 | 19/28 | 14/16 | 13/14 |
| FF∆2bL | 13/22 | 20/28 | 16/16 | 14/14 |
| FL∆2bF | 9/22 | 24/28 | 13/16 | 13/14 |
| LF∆2bF | 22/22 | 26/28 | 16/16 | 14/14 |
| **B. Total aphid offspring at 14 dpi** | | | | |
| Mock-Inoculated | 150 | 404 | 287 | 152 |
| Fny-CMV | 470 | 638 | 451 | 258 |
| Fny-CMV∆2b | 66 | 238 | 156 | 43 |
| FF∆2bL | 61 | 201 | 202 | 37 |
| FL∆2bF | 56 | 250 | 183 | 48 |
| LF∆2bF | 240 | 472 | 313 | 129 |
| **C. Mean offspring per founder aphid at 14 dpi** | | | | |
| Mock-Inoculated | 6.8 | 14.4 | 17.9 | 10.9 |
| Fny-CMV | 21.4 | 22.8 | 28.2 | 18.4 |
| Fny-CMV∆2b | 3 | 8.5 | 9.8 | 3.1 |
| FF∆2bL | 2.8 | 7.2 | 13.2 | 2.6 |
| FL∆2bF | 2.5 | 9 | 12.5 | 3.7 |
| LF∆2bF | 10.9 | 17.9 | 21.2 | 9.2 |

**Notes.** Aphid survival and offspring from four independent experiments. Statistical analysis for the aphid reproduction is described in Table S4 below. Data from Experiment 2 are displayed as a bar chart in Fig. 2.
