# Supplementary material for: Cucumber mosaic virus 2b proteins inhibit virus‐induced aphid resistance in tobacco
Source: Mol Plant Pathol. 2019 Nov 27;21(2):250–7. doi: 10.1111/mpp.12892 (PMC6988427; doi:10.1111/mpp.12892)
Supplement: Supplementary file 6 — Table S4 Statistical analysis on aphid reproduction on tobacco plants infected with wild‐type Fny‐CMV, Fny‐CMVΔ2b LS‐CMV and reassortant viruses lacking the ability to express the 2b gene. [file MPP-21-250-s006.docx]

**Table S4.** Statistical analysis on aphid reproduction on tobacco plants infected with wild-type Fny-CMV, Fny-CMV∆2b LS-CMV and reassortant viruses lacking the ability to express the *2b* gene.

| **Experiment 1** | Total aphid offspring | Mock | Fny-CMV | Fny-CMV∆2b | FF∆2bL | FL∆2bF | LF∆2bF |
| --- | --- | --- | --- | --- | --- | --- | --- |
|  |  | 150 | 470 | 66 | 61 | 56 | 240 |
| Mock | 150 |  |  |  |  |  |  |
| Fny-CMV | 470 | * |  |  |  |  |  |
| Fny-CMV∆2b | 66 |  | * |  |  |  |  |
| FF∆2bL | 61 | * | * |  |  |  |  |
| FL∆2bF | 56 |  | * |  |  |  |  |
| LF∆2bF | 240 |  | * | * | * | * |  |
|  | | | | | | | |
| **Experiment 2** | Total aphid offspring | Mock | Fny-CMV | Fny-CMV∆2b | FF∆2bL | FL∆2bF | LF∆2bF |
|  |  | 404 | 638 | 238 | 201 | 250 | 472 |
| Mock | 404 |  |  |  |  |  |  |
| Fny-CMV | 638 | * |  |  |  |  |  |
| Fny-CMV∆2b | 238 |  | * |  |  |  |  |
| FF∆2bL | 201 | * | * |  |  |  |  |
| FL∆2bF | 250 | * | * |  |  |  |  |
| LF∆2bF | 472 |  |  | * | * | * |  |
|  | | | | | | | |
| **Experiment 3** | Total aphid offspring | Mock | Fny-CMV | Fny-CMV∆2b | FF∆2bL | FL∆2bF | LF∆2bF |
|  |  | 287 | 451 | 156 | 202 | 183 | 313 |
| Mock | 287 |  |  |  |  |  |  |
| Fny-CMV | 451 | * |  |  |  |  |  |
| Fny-CMV∆2b | 156 | * | * |  |  |  |  |
| FF∆2bL | 202 | * | * |  |  |  |  |
| FL∆2bF | 183 |  | * |  |  |  |  |
| LF∆2bF | 313 |  | * | * | * | * |  |
|  | | | | | | | |
| **Experiment 4** | Total aphid offspring | Mock | Fny-CMV | Fny-CMV∆2b | FF∆2bL | FL∆2bF | LF∆2bF |
|  |  | 152 | 258 | 43 | 37 | 48 | 129 |
| Mock | 152 |  |  |  |  |  |  |
| Fny-CMV | 258 | * |  |  |  |  |  |
| Fny-CMV∆2b | 43 | * | * |  |  |  |  |
| FF∆2bL | 37 | * | * |  |  |  |  |
| FL∆2bF | 48 | * | * |  |  |  |  |
| LF∆2bF | 129 |  | * | * | * | * |  |
|  | | | | | | | |
| Across all experiments | Total aphid offspring | Mock | Fny-CMV | Fny-CMV∆2b | FF∆2bL | FL∆2bF | LF∆2bF |
|  |  |  |  |  |  |  |  |
| Mock |  |  |  |  |  |  |  |
| Fny-CMV |  | * |  |  |  |  |  |
| Fny-CMV∆2b |  | * | * |  |  |  |  |
| FF∆2bL |  | * | * |  |  |  |  |
| FL∆2bF |  | * | * |  |  |  |  |
| LF∆2bF |  |  | * | * | * | * |  |

**Notes.** Negative binomial regression (as explained above) were used to analyse statistical significance for pairwise comparisons made between treatments on aphid reproduction. Pairwise comparison marked with * denotes significance at *p* < 0.05 with FDR-adjusted *p-*value.

Aphids confined on Fny-CMV-infected plants gave rise to significantly more offspring compared to aphids confined on mock-infected plants and on plants infected with the 2b mutant viruses respectively (Fny-CMV∆2b, FF∆2bL, FL∆2bF) in all four experiments. Also in all four experiments, aphids confined on LF∆2bF-infected tobacco plants produced significantly more offspring compared to aphids on Fny-CMV∆2b-infected plants. Data from Experiment 2 are displayed as a bar chart in Fig. 2.
