# Supplementary material for: Cucumber mosaic virus 2b proteins inhibit virus‐induced aphid resistance in tobacco
Source: Mol Plant Pathol. 2019 Nov 27;21(2):250–7. doi: 10.1111/mpp.12892 (PMC6988427; doi:10.1111/mpp.12892)
Supplement: Supplementary file 7 — Table S5 Aphid performance on tobacco plants infected with Fny‐CMV, Fny‐CMVΔ2b LS‐CMV, selected reassortant viruses including those constituted using a recombinant Fny‐CMV RNA 2 expressing the LS‐CMV 2b gene sequence. [file MPP-21-250-s007.docx]

**Table S5.** Aphid performance on tobacco plants infected with Fny-CMV, Fny-CMV∆2b LS-CMV, selected reassortant viruses including those constituted using a recombinant Fny-CMV RNA 2 expressing the LS-CMV *2b* gene sequence.

| **A. Number of surviving founder aphids at 14 dpi** | | | |
| --- | --- | --- | --- |
| **Treatment** | **Exp 1 (n = 12-14)** | **Exp 2 (n=14)** | **Exp 3 (n=12)** |
| Mock-Inoculated | 12/12 | 14/14 | 12/12 |
| Fny-CMV | 12/12 | 14/14 | 12/12 |
| Fny-CMV∆2b | 9/12 | 9/14 | 10/12 |
| FFL | 11/14 | 14/14 | 12/12 |
| FLF | 12/14 | 14/14 | 12/12 |
| LFF | 11/12 | 13/14 | 12/12 |
| FF(L2b)F | 12/13 | 14/14 | 12/12 |
| LF(L2b)F | 12/12 | 13/14 | 12/12 |
| **B. Total aphid offspring at 14 dpi** | | | |
| Mock-Inoculated | 200 | 226 | 163 |
| Fny-CMV | 350 | 389 | 312 |
| Fny-CMV∆2b | 85 | 59 | 82 |
| FFL | 280 | 272 | 291 |
| FLF | 281 | 305 | 283 |
| LFF | 272 | 295 | 306 |
| FF(L2b)F | 255 | 292 | 275 |
| LF(L2b)F | 271 | 302 | 296 |
| **C. Mean offspring per founder aphid at 14 dpi** | | | |
| Mock-Inoculated | 16.7 | 16.1 | 13.6 |
| Fny-CMV | 29.2 | 27.8 | 26 |
| Fny-CMV∆2b | 7.1 | 4.2 | 6.8 |
| FFL | 20 | 19.4 | 24.3 |
| FLF | 20.1 | 21.8 | 23.6 |
| LFF | 22.7 | 21.1 | 25.5 |
| FF(L2b)F | 19.6 | 20.9 | 22.9 |
| LF(L2b)F | 22.6 | 21.6 | 24.7 |

**Notes.** Aphid survival and offspring from four independent experiments. Statistical analysis for aphid reproduction is described in Table S6 below. Data from Experiment 3 are displayed as a bar chart in Fig. 3.
