# Supplementary material for: Cucumber mosaic virus 2b proteins inhibit virus‐induced aphid resistance in tobacco
Source: Mol Plant Pathol. 2019 Nov 27;21(2):250–7. doi: 10.1111/mpp.12892 (PMC6988427; doi:10.1111/mpp.12892)
Supplement: Supplementary file 8 — Table S6 Statistical analysis for aphid reproduction on tobacco plants infected with Fny‐CMV, Fny‐CMVΔ2b LS‐CMV, selected reassortant viruses including those constituted using a recombinant Fny‐CMV RNA 2 expressing the LS‐CMV 2b gene sequence. [file MPP-21-250-s008.docx]

**Table S6.** Statistical analysis for aphid reproduction on tobacco plants infected with Fny-CMV, Fny-CMV∆2b LS-CMV, selected reassortant viruses including those constituted using a recombinant Fny-CMV RNA 2 expressing the LS-CMV *2b* gene sequence.

| **Experiment 1** | Total aphid offspring | Mock | Fny-CMV | Fny-CMV∆2b | FFL | FLF | LFF | FF(L2b)F | LF(L2b)F |
| --- | --- | --- | --- | --- | --- | --- | --- | --- | --- |
|  |  | 200 | 350 | 85 | 280 | 281 | 272 | 255 | 271 |
| Mock | 200 |  |  |  |  |  |  |  |  |
| Fny-CMV | 350 | * |  |  |  |  |  |  |  |
| Fny-CMV∆2b | 85 | * | * |  |  |  |  |  |  |
| FFL | 280 |  | * | * |  |  |  |  |  |
| FLF | 281 |  | * | * |  |  |  |  |  |
| LFF | 272 | * |  | * |  |  |  |  |  |
| FF(L2b)F | 255 |  | * | * |  |  |  |  |  |
| LF(L2b)F | 271 | * | * | * |  |  |  |  |  |
|  | | | | | | | | | |
| **Experiment 2** | Total aphid offspring | Mock | Fny-CMV | Fny-CMV∆2b | FFL | FLF | LFF | FF(L2b)F | LF(L2b)F |
|  |  | 226 | 389 | 59 | 272 | 305 | 295 | 292 | 301 |
| Mock | 226 |  |  |  |  |  |  |  |  |
| Fny-CMV | 389 | * |  |  |  |  |  |  |  |
| Fny-CMV∆2b | 59 | * | * |  |  |  |  |  |  |
| FFL | 272 | * | * | * |  |  |  |  |  |
| FLF | 305 | * | * | * |  |  |  |  |  |
| LFF | 295 | * | * | * |  |  |  |  |  |
| FF(L2b)F | 292 | * | * | * |  |  |  |  |  |
| LF(L2b)F | 301 | * | * | * |  |  |  |  |  |
|  | | | | | | |  |  |  |
| **Experiment 3** | Total aphid offspring | Mock | Fny-CMV | Fny-CMV∆2b | FFL | FLF | LFF | FF(L2b)F | LF(L2b)F |
|  |  | 163 | 312 | 82 | 291 | 283 | 306 | 275 | 296 |
| Mock | 163 |  |  |  |  |  |  |  |  |
| Fny-CMV | 312 | * |  |  |  |  |  |  |  |
| Fny-CMV∆2b | 82 | * | * |  |  |  |  |  |  |
| FFL | 291 | * |  | * |  |  |  |  |  |
| FLF | 283 | * |  | * |  |  |  |  |  |
| LFF | 306 | * |  | * |  |  |  |  |  |
| FF(L2b)F | 275 | * |  | * |  |  |  |  |  |
| LF(L2b)F | 296 | * |  | * |  |  |  |  |  |
|  | | | | | | |  |  |  |
| Across all experiments | Total aphid offspring | Mock | Fny-CMV | Fny-CMV∆2b | FFL | FLF | LFF | FF(L2b)F | LF(L2b)F |
|  |  |  |  |  |  |  |  |  |  |
| Mock |  |  |  |  |  |  |  |  |  |
| Fny-CMV |  | * |  |  |  |  |  |  |  |
| Fny-CMV∆2b |  | * | * |  |  |  |  |  |  |
| FFL |  | * | * | * |  |  |  |  |  |
| FLF |  | * | * | * |  |  |  |  |  |
| LFF |  | * |  | * |  |  |  |  |  |
| FF(L2b)F |  | * | * | * |  |  |  |  |  |
| LF(L2b)F |  | * | * | * |  |  |  |  |  |

**Notes.** Negative binomial regression (as explained above) were used to analyse statistical significance for pairwise comparisons made between treatments on aphid reproduction. Pairwise comparison marked with * denotes significance at *p* < 0.05 with FDR-adjusted *p-*value.

Aphids (*Myzus persicae*) confined on tobacco plants infected with Fny-CMV∆2b gave rise to significantly fewer offspring compared to aphids placed on plants under other treatment conditions (mock-inoculated, infected with Fny-CMV, or other reassortant/recombinant viruses) in all three experiments here. This finding is consistent with findings in Tables S1 and S3

Aphids confined on tobacco plants infected with Fny-CMV gave rise to significantly more offspring compared to aphids on mock-inoculated plants in all three experiments.

In Experiment 2 and 3, aphids confined on plants infected with the five reassortant viruses respectively (with the exception of aphids on Fny-CMV∆2b-infected plants) gave rise to significantly more offspring compared to aphids confined on mock-inoculated plants. Data from Experiment 3 are displayed as a bar chart in Fig. 3.
