# Supplementary material for: Cucumber mosaic virus 2b proteins inhibit virus‐induced aphid resistance in tobacco
Source: Mol Plant Pathol. 2019 Nov 27;21(2):250–7. doi: 10.1111/mpp.12892 (PMC6988427; doi:10.1111/mpp.12892)
Supplement: Supplementary file 9 — Methods S1 Supplementary Methods. [file MPP-21-250-s009.docx]

**Supplementary Methods**

**Analysis of aphid survival on tobacco plants infected with wild-type Fny-CMV and mutant variants**

Data from aphid survival experiments described in Fig. 4 were analyzed using binomial logistic regression. There were systematic differences in overall survival rates of aphids between experiments. This is caused by intrinsic variation in different batches of aphids and plants used for each experimental repeats. We accounted for this by including the identity of the experiment as a blocking factor in our analyses. The data from the *i*^th^ experiment consisted of counts of the number of surviving (*s_ij_*) and dead (*d_ij_*) aphids on plants of the *j*^th^ treatment (i.e. infection with a particular virus), where in general *s_ij_* + *d_ij_* is smaller than the initial number of aphids used for each treatment (n=50) since some aphids died over the course of the experiment.

We fitted the following model for the probability of aphids surviving until the 11^th^ day post-placement to these data

in which the intercept $\alpha_{0}$ is the logarithm of the odds ratio of aphid survival on mock-inoculated plants, $\alpha_{j}$is the effect of the *j*^th^ treatment (virus type) and $\beta_{i}$is the nuisance effect of the *i*^th^ experiment. Statistical significance of the *j*^th^ treatment was then assessed by considering the 95% confidence interval on the treatment effect parameter $\alpha_{j}$, i.e. the logarithm of the odds ratio of the *j*^th^ treatment relative to mock-inoculated plants.

**Analysis of aphid reproduction on tobacco plants infected with wild-type FNY-CMV and mutant variants**

Data from aphid reproduction described in Figs 1-3 and Table S1, S3, and S5 below were analysed using negative binomial regression. There were systematic differences in overall reproduction rates of aphids between batches. This is caused by intrinsic variation in different batches of aphids and plants used for each experimental repetition. We took account of this in two different ways.

Firstly, we performed regression analysis at the batch scale, but with a correction that accounts for the multiple batches that comprise a particular experiment. In this way, contrasts between treatments were tested for statistical significance using negative binomial glm models with survival (since the survival of founder aphids in part determines offspring produced) and treatment included as fixed effects (using the *multcomp* package in R) (Hothorn *et al.*, 2008), in which the response variable was the number of aphid nymphs on a plant after 14 days (and where a single aphid was confined to a cage on day 0, and left to reproduce for 14 days). For a given experiment in which there were $m$ treatments repeated in $n$ batches, we extracted $m\left( m-1 \right)/2$ pairwise contrasts for each batch. We then collected the contrasts for each batch to produce a total of $nm\left( m-1 \right)/2$ contrasts. We performed a correction for multiple contrasts across all $nm\left( m-1 \right)/2$ contrasts using the Benjamani-Hochberg procedure to control the type $1$ error rate. The Benjamani-Hochberg procedure is a false discovery rate correction and is appropriate to use instead of familywise error rate corrections (e.g., Bonferroni correction) in situations with a large number of contrasts (Benjamini and Hochberg, 1995). As this approach corrects simultaneously across batches in a particular experiment, it allows us to discuss results at the level of experiments by reference to significance within batches (e.g. in three out of five batches in experiment 1, *FNY-CMV*$\Delta$*2b* virus inoculated tobacco plants lead to increased aphid reproduction relative to mock inoculated plants). Batches here referred to the number of times the experiment have been repeated independently.

Secondly, to complement both the above approach and the analysis of aphid survival (Analysis of aphid survival on tobacco plants infected with wild-type Fny-CMV and mutant variants), we accounted for batch variation by performing a *regression analysis across all experiments* including the identity of the batch as a blocking factor in a negative binomial glm together with the fixed effects, survival and virus treatment. In this case, i.e., the *regression analysis across all experiments,* since the number of contrasts was small (i.e., $m\left( m-1 \right)/2$ instead of $nm\left( m-1 \right)/2$) we performed a Bonferroni correction. Note that these results are included only to support the analyses at the batch scale and hence are not referenced in the main text (see final parts of Tables S2, S4 and S6). All analyses were conducted in R version 3.5.0 (R: A language and environment for statistical computing. R 477 Foundation for Statistical Computing, Vienna, Austria. Available online at 478 https://www.R-project.org/).

**Double antibody sandwich enzyme-linked immunosorbent assay (DAS ELISA) to verify viral infection**

At 14 days post-inoculation, accumulation of CMV CP was measured by DAS-ELISA from leaf samples taken from non-inoculated leaves. Leaf discs were harvested using a 1.5 cm diameter cork borer and processed according to the manufacturers protocols for DAS-ELISA (Bioreba AG). Absorbance was measured at 30 minutes after p-nitrophenyl phosphate is added to each well, using a plate reader at 405 nm. The A^405^ value from the mock-inoculated plant was subtracted to give the corrected absorbance.
